# Exogenous spermidine is enhancing tomato tolerance to salinity–alkalinity stress by regulating chloroplast antioxidant system and chlorophyll metabolism

**DOI:** 10.1186/s12870-015-0699-7

**Published:** 2015-12-29

**Authors:** Jianming Li, Lipan Hu, Li Zhang, Xiongbo Pan, Xiaohui Hu

**Affiliations:** College of Horticulture, Northwest A&F University, Yangling, 712100 Shaanxi China; Key Laboratory of Protected Horticultural Engineering in Northwest, Ministry of Agriculture, Yangling, 712100 Shaanxi China

**Keywords:** Spermidine, Tomato, Salinity–alkalinity stress, Chloroplast, Chlorophyll precursor, Antioxidant system

## Abstract

**Background:**

Salinity–alkalinity stress is known to adversely affect a variety of processes in plants, thus inhibiting growth and decreasing crop yield. Polyamines protect plants against a variety of environmental stresses. However, whether exogenous spermidine increases the tolerance of tomato seedlings via effects on chloroplast antioxidant enzymes and chlorophyll metabolism is unknown. In this study, we examined the effect of exogenous spermidine on chlorophyll synthesis and degradation pathway intermediates and related enzyme activities, as well as chloroplast ultrastructure, gene expression, and antioxidants in salinity–alkalinity–stressed tomato seedlings.

**Results:**

Salinity–alkalinity stress disrupted chlorophyll metabolism and hindered uroorphyrinogen III conversion to protoporphyrin IX. These effects were more pronounced in seedlings of cultivar Zhongza No. 9 than cultivar Jinpengchaoguan. Under salinity–alkalinity stress, exogenous spermidine alleviated decreases in the contents of total chlorophyll and chlorophyll a and b in seedlings of both cultivars following 4 days of stress. With extended stress, exogenous spermidine reduced the accumulation of δ–aminolevulinic acid, porphobilinogen, and uroorphyrinogen III and increased the levels of protoporphyrin IX, Mg–protoporphyrin IX, and protochlorophyllide, suggesting that spermidine promotes the conversion of uroorphyrinogen III to protoporphyrin IX. The effect occurred earlier in cultivar Jinpengchaoguan than in cultivar Zhongza No. 9. Exogenous spermidine also alleviated the stress–induced increases in malondialdehyde content, superoxide radical generation rate, chlorophyllase activity, and expression of the chlorophyllase gene and the stress–induced decreases in the activities of antioxidant enzymes, antioxidants, and expression of the porphobilinogen deaminase gene. In addition, exogenous spermidine stabilized the chloroplast ultrastructure in stressed tomato seedlings.

**Conclusions:**

The tomato cultivars examined exhibited different capacities for responding to salinity–alkalinity stress. Exogenous spermidine triggers effective protection against damage induced by salinity–alkalinity stress in tomato seedlings, probably by maintaining chloroplast structural integrity and alleviating salinity–alkalinity–induced oxidative damage, most likely through regulation of chlorophyll metabolism and the enzymatic and non–enzymatic antioxidant systems in chloroplast. Exogenous spermidine also exerts positive effects at the transcription level, such as down–regulation of the expression of the chlorophyllase gene and up–regulation of the expression of the porphobilinogen deaminase gene.

## Background

Tomato (*Solanum lycopersicum* L.) is one of the most widely cultivated vegetables in the world. However, tomato production is negatively impacted by soil salinization and alkalization, which frequently co–occur in nature and are some of the most adverse environmental stresses to plants and tomato in particular [1, 2]. Salinity–alkalinity stress is known to adversely affect a variety of processes in plants, such as seed germination, ion uptake, stomata opening, and photosynthetic rate [3]. Our previous study showed that salinity–alkalinity stress decreases tomato growth, nitrogen metabolism [1], polyamine metabolism [4], and photosynthetic efficiency, which significantly impacts the growth and development of plants.

Chlorophyll (Chl) receives solar energy in photosynthetic antenna systems and mediates charge separation and electron transport within reaction centers [5]. Chl is essential for light harvesting and energy transduction in photosynthesis. The Chl content determines photosynthesis, which in turn determines plant growth and development. The level of Chl is maintained by a balance between Chl biosynthesis and degradation [6, 7]. Previous research has found that salt stress disturbs the balance between Chl biosynthesis and degradation, thus altering the Chl content [8]. The Chl synthesis pathway is mediated by more than 17 enzymes [9]. Blockade of any step in the chlorophyll biosynthesis pathway will cause a decline in Chl content. Chlorophyllase (Chlase) plays an important role in chlorophyll degradation. Regulation of the levels of Chl and its derivatives, such as protochlorophyll (Pchl) and protoporphyrin IX (Proto IX), is extremely important, because these molecules are strong photosensitizers; that is, when present in excess, they will generate reactive oxygen species (ROS) [10]. ROS, in turn, may retard cell growth or even cause cell death. Therefore, to maintain healthy growth, plants must exert fine control over the entire Chl metabolic process. Sun et al. reported that in spinach cultivars undergoing seawater stress, the levels of Chl b, Chl a, total Chl decreased significantly [10]. The decreased chlorophyll may attribute to accumulate much more ROS in chloroplast. ROS hinders the transformation of porphobilinogen (PBG) to uroorphyrinogen III (URO III) [10].

The accumulation of ROS is a general feature of salinity stress that alters the antioxidation capacity of cells, leading to oxidative damage [11] as well as ROS signaling [12]. Chloroplasts are major sites of ROS generation under stress conditions [13]. To counteract the toxicity of ROS, plants have highly efficient antioxidative systems composed of both nonenzymatic antioxidants and antioxidant enzymes. The non–enzymatic antioxidants include ascorbate (AsA), glutathione (GSH), carotenoids, flavanones, and anthocyanins, whereas antioxidant enzymes include superoxide dismutase (SOD), catalase (CAT), ascorbate peroxidase (APX), monodehydroascorbate reductase (MDHAR), dehydroascorbate reductase (DHAR), glutathione reductase (GR), glutathione peroxidase (GPX), and glutathione *S*–transferase [14]. It has been hypothesized that the accumulation of ROS in chloroplasts due to salinity–alkalinity stress can be mitigated by enhancing the antioxidant capacity [2]. The ascorbate–glutathione cycle appears to play an important role in maintaining the redox status in plant cells, especially under abiotic stress [15].

Polyamines are a class of biogenic amines that exert multiple *in vivo* effects on cellular processes in most organisms [16]. Considerable research indicates that polyamines play an important role in protecting plants against abiotic stress [17, 18]. Compared with other polyamines (PAs), spermidine (Spd) more effectively alleviates the adverse effects of salinity–alkalinity stress [4]. We found that exogenous Spd treatment can regulate the metabolic status of polyamines caused by salinity–alkalinity stress, and eventually enhance tolerance of tomato plants to salinity–alkalinity stress [4]. PAs catabolism is tightly linked to ROS generation, because amino oxidases generate hydrogen peroxide (H_2_O_2_), which mediates ROS signaling [19]. In a previous study, we found that exogenous Spd can alleviate the decrease of root dry weight caused by salinity–alkalinity stress [4]. However, whether a close relationship exists between exogenous Spd and increased stress tolerance in tomato seedlings due to induction of antioxidant enzymes and altered chlorophyll metabolism in chloroplasts is unclear.

In this study, we examined the effects of exogenous Spd on the antioxidant system in chloroplasts in salinity–alkalinity–stressed tomato seedlings. We also examined the effects of exogenous Spd on the Chl synthesis and degradation pathways to evaluate the role of exogenous Spd in Chl metabolism. Specifically, we examined the levels of Chl and related molecules, the activities of various enzymes, the expression of relevant genes, and changes in chloroplast ultrastructure. The overall objective of the present study was to elucidate the mechanism of Spd–mediated protection of the photochemical pathways and structures from salinity–alkalinity–induced damage in tomato seedlings.

We found that exogenous Spd is effective in triggering protection against cellular and macromolecular damage in tomato seedlings during salinity–alkalinity stress. Exogenous Spd showed positive effects on maintaining the structural integrity of chloroplasts. This may be because exogenous Spd alleviate salinity–alkalinity–induced oxidative damage, through regulation of Chl metabolism and enzymatic and non–enzymatic antioxidant systems in the chloroplasts.

## Results

### The impact of Spd on Chl content in salinity–alkalinity–stressed tomato seedlings

**Fig. 1:**
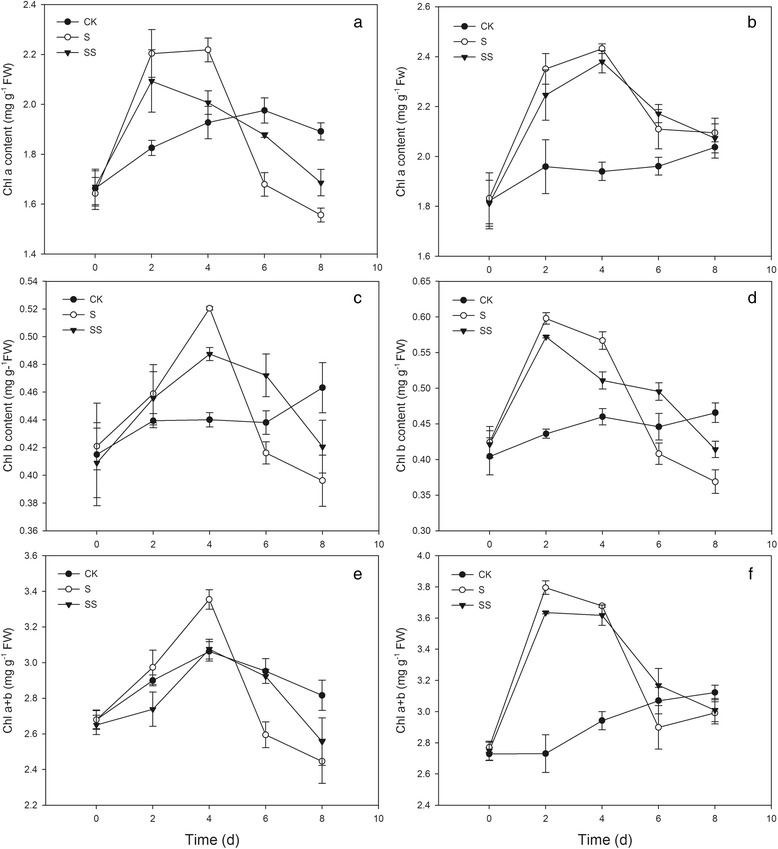
Effect of exogenous Spd on chlorophyll content in tomato seedlings. CK, 1/2 Hoagland’s solution; S, 75 mM saline–alkaline solution (NaCl: Na_2_SO_4_: NaHCO_3_: Na_2_CO_3_ = 1:9:9:1); SS, sprayed with 0.25 mM Spd and treated with 75 mM saline–alkaline solution. **a, c** and **e** represent cv. Zhongza No.9; (**b, d** and **f)** represent cv. Jinpengchaoguan

As shown in Fig. 1, the contents of Chl a, Chl b and total Chl in salinity–alkalinity–stress (S)–treated two tomato cultivars increased early and decreased later, and peaked on fourth day, except for Chl b and total Chl contents in cv. Jinpengchaoguan (cv. JP) peaked on the second day. Compared with the control, the Chl content trended upward for 4 days after the initiation of salinity–alkalinity conditions, but then the levels declined and became significantly lower compared with CK–treated plants. During salinity–alkalinity stress, this trend was suppressed to some extent by salinity–alkalinity plus Spd (SS) treatment, as after 4 days of SS treatment, the decreases in Chl a, Chl b, and total Chl content in stressed seedlings of both cultivars were alleviated (Fig. 1).

### Effect of Spd on Chl precursor content in salinity–alkalinity–stressed tomato seedlings

**Fig. 2:**
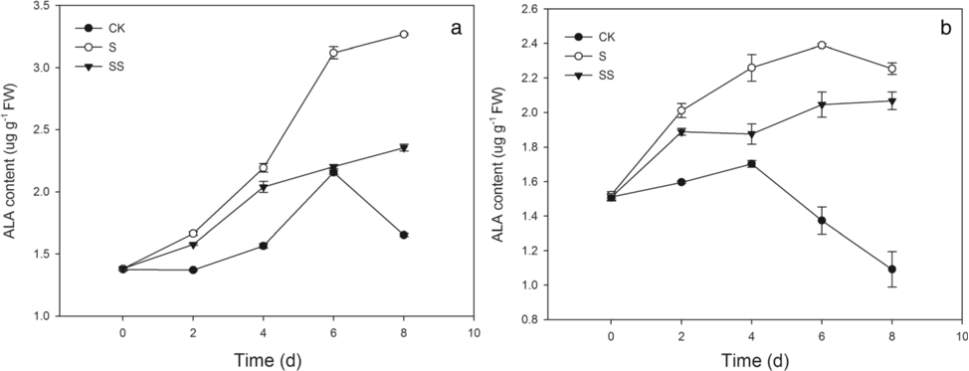
Effect of Spd on ALA content in tomato seedlings. CK, 1/2 Hoagland’s solution; S, 75 mM saline–alkaline solution (NaCl: Na_2_SO_4_: NaHCO_3_: Na_2_CO_3_ = 1:9:9:1); SS, sprayed with 0.25 mM Spd and treated with 75 mM saline–alkaline solution. **a** represents cv. Zhongza No.9; **b** represents cv. Jinpengchaoguan

The level of ALA (δ–aminolevulinic acid) in both cultivars under CK conditions rose during the early period of treatment and then decreased, peaking on day 6 and day 4 after treatment in cv. Zhongza No.9 (cv. ZZ) and cv. JP, respectively. ALA levels in S–treated seedlings were significantly higher than in CK–treated seedlings in both cultivars. However, exogenous Spd significantly reduced the stress–induced increase in ALA level. In addition, cv. JP had higher ALA levels than cv. ZZ during treatment days 0 to 4, but after day 4, cv. JP had lower ALA levels than cv. ZZ (Fig. 2).

**Fig. 3:**
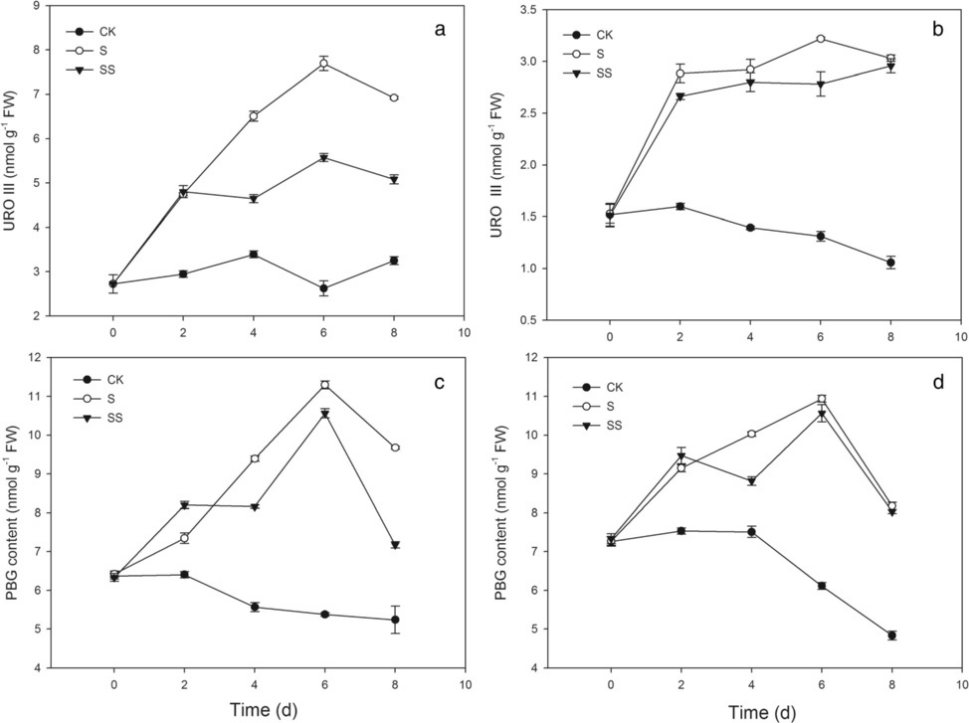
Effect of Spd on URO III and PBG content in tomato seedlings. CK, 1/2 Hoagland’s solution; S, 75 mM saline–alkaline solution (NaCl: Na_2_SO_4_: NaHCO_3_: Na_2_CO_3_ = 1:9:9:1); SS, sprayed with 0.25 mM Spd and treated with 75 mM saline–alkaline solution. **a** and **c** represent cv. Zhongza No.9; **b** and **d** represent cv. Jinpengchaoguan

The PBG and uroorphyrinogen III (URO III) contents in both cultivars grown under CK conditions exhibited a similar but slightly different trend as ALA (Fig. 3). Under salinity–alkalinity stress, the PBG content significantly increased and peaked on treatment day 6. The stress–induced accumulation of PBG was alleviated by exogenous Spd in cv. ZZ. Stress also caused significant increase in the URO III content in both cv. ZZ and cv. JP after treatment day 2, peaking on day 6 (Fig. 3). SS treatment reduced the stress–induced increase in URO III content. In addition, cv. JP had higher PBG content and lower URO III content than cv. ZZ under the same treatment conditions (Fig. 3).

**Fig. 4:**
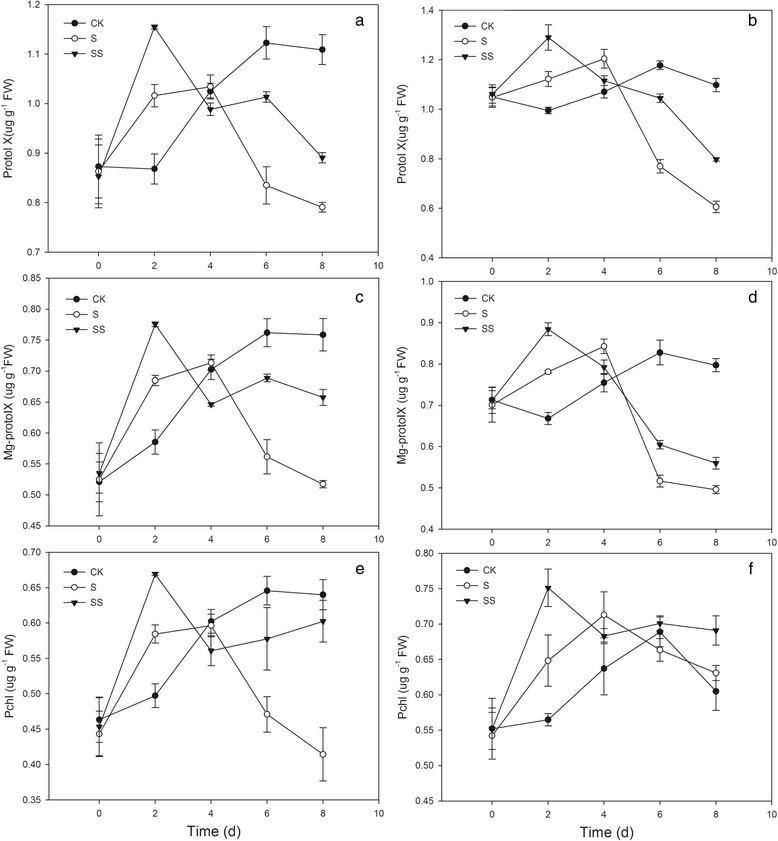
Effect of Spd on Proto IX, Mg–proto IX and Pchl content in tomato seedlings. CK, 1/2 Hoagland’s solution; S, 75 mM saline–alkaline solution (NaCl: Na_2_SO_4_: NaHCO_3_: Na_2_CO_3_ = 1:9:9:1); SS, sprayed with 0.25 mM Spd and treated with 75 mM saline–alkaline solution. **a, c** and **e** represent cv. Zhongza No.9; **b, d** and **f**) represent Jinpengchaoguan

Under salinity–alkalinity stress, the Proto IX and Mg–Proto IX contents in both cultivars exhibited similar changes, rising early but declining later, with maximum levels occurring on day 4 (Fig. 4). Compared with S treatment, SS treatment led to a significant increase in the Proto IX content, except on day 6. SS treatment also significantly increased the Mg–Proto IX and Pchl levels, except on day 4 (Fig. 4).

### Effect of Spd on Chlase activity in salinity–alkalinity–stressed tomato seedlings

**Fig. 5:**
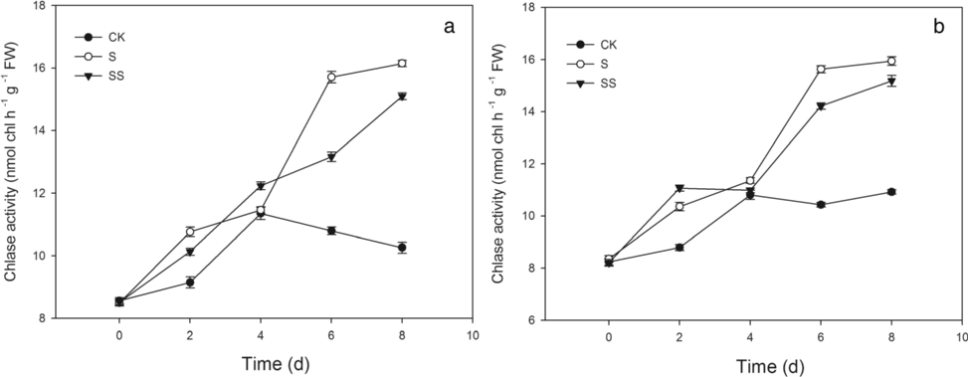
Effect of Spd on Chlase activity in tomato seedlings. CK, 1/2 Hoagland’s solution; S, 75 mM saline–alkaline solution (NaCl: Na_2_SO_4_: NaHCO_3_: Na_2_CO_3_ = 1:9:9:1); SS, sprayed with 0.25 mM Spd and treated with 75 mM saline–alkaline solution. **a** represents cv. Zhongza No.9; **b** represents cv. Jinpengchaoguan

Under CK conditions, Chlase activity remained relatively stable and low in both cultivars (Fig. 5). An increase in Chlase activity was evident on the second day after exposure to salinity–alkalinity stress. With the exception of day 4 for cv. ZZ and day 2 for cv. JP, the Chlase activity in both cultivars was higher with S treatment than with SS treatment. Throughout the stress period, no obvious difference was observed in Chlase activity in SS–treated cv. ZZ and cv. JP seedlings.

### Effect of Spd on Malondialdehyde (MDA) content and O_2_^•−^ generation rate in salinity–alkalinity–stressed tomato seedlings chloroplasts

**Fig. 6:**
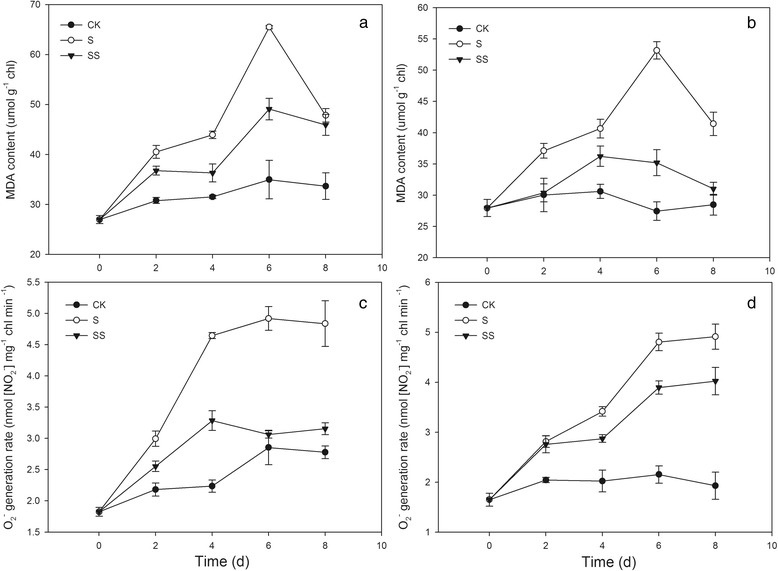
Effect of Spd on MDA content and O_2_^–⋅^ generation rate in tomato seedlings. CK, 1/2 Hoagland’s solution; S, 75 mM saline–alkaline solution (NaCl: Na_2_SO_4_: NaHCO_3_: Na_2_CO_3_ = 1:9:9:1); SS, sprayed with 0.25 mM Spd and treated with 75 mM saline–alkaline solution. **a** and **c** represent cv. Zhongza No.9; **b** and **d** represent cv. Jinpengchaoguan

MDA is the final product of lipid peroxidation, and the MDA level increased in the chloroplasts of both tomato cultivars under stress conditions compare with CK treatment, reaching the highest level on day 6 (Fig. 6). Under salinity–alkalinity stress with application of exogenous Spd, the MDA content in the chloroplasts was significantly reduced in both cultivars. 6 days after treatment, compared with S treatment, MDA content in SS treatment of plants decreased by 25.01 % (for cv. Zhongza No.9) and 33.79 % (for cv. Jinpengchaoguan), respectively (Fig. 6).

ROS levels are indicators of stress in plants. The rate of O_2_^•−^ generation was higher in the chloroplasts of stressed tomato seedlings compared with CK–treated seedlings, and the rate was higher in cv. JP than in cv. ZZ during the experimental period, except on day 4 (Fig. 6). However, the O_2_^•−^ generation rate was significantly lower in the chloroplasts of SS–treated seedlings of both cultivars subjected to salinity–alkalinity stress. Furthermore, the amplitude of the change in O_2_^•−^ generation rate was higher in cv. ZZ than in cv. JP when seedlings were treated with exogenous Spd under conditions of salinity–alkalinity stress (Fig. 6).

### Effect of Spd on the chloroplast antioxidant system of salinity–alkalinity–stressed tomato seedlings

**Fig. 7:**
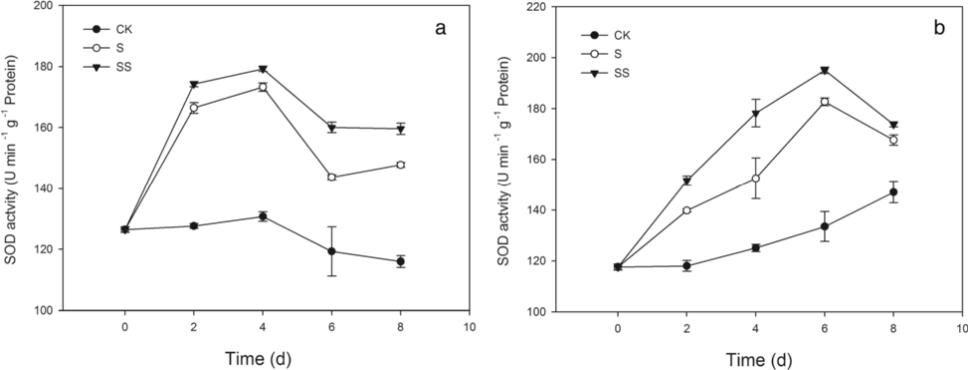
Effect of exogenous Spd on SOD activity in tomato seedlings. CK, 1/2 Hoagland’s solution; S, 75 mM saline–alkaline solution (NaCl:Na_2_SO_4_:NaHCO_3_:Na_2_CO_3_ = 1:9:9:1); SS, sprayed with 0.25 mM Spd and treated with 75 mM saline–alkaline solution. **a** represents cv. Zhongza No. 9; **b** represents cv. Jinpengchaoguan

**Fig. 8:**
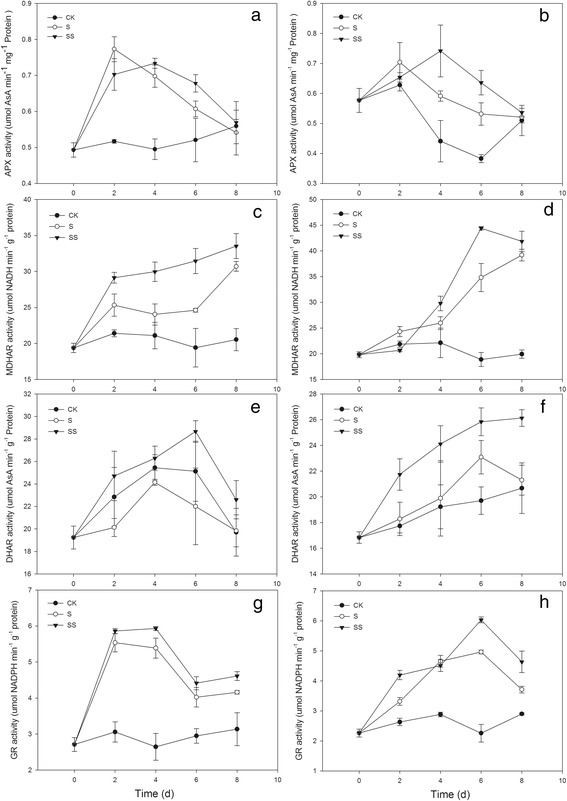
Effect of exogenous Spd on APX, MDHAR, DHAR and GR activity in tomato seedlings. CK, 1/2 Hoagland’s solution; S, 75 mM saline–alkaline solution (NaCl: Na_2_SO_4_: NaHCO_3_: Na_2_CO_3_ = 1:9:9:1); SS, sprayed with 0.25 mM Spd and treated with 75 mM saline–alkaline solution. **a, c, e** and **g** represent cv. Zhongza No.9; (**b, d, f** and **h**) represent cv. Jinpengchaoguan

The activities of superoxide dismutase (SOD), ascorbate peroxidase (APX), and glutathione reductase (GR) increased significantly in chloroplasts of seedlings of the both tomato cultivars during exposure to salinity–alkalinity stress, peaking on day 2 in cv. ZZ seedlings and on days 6, 4, and 6, in cv. JP seedlings, respectively (Figs. 7 and 8). The monodehydroascorbate reductase (MDHAR) activity in the chloroplasts of stressed tomato seedlings of both cultivars was significantly higher than that of CK–treated seedlings (Fig. 8). Compared with CK–treated seedlings, those subjected to salinity–alkalinity stress exhibited significantlly reduced dehydroascorbate reductase (DHAR) activity in cv. ZZ and increased DHAR activity in cv. JP (Fig. 8). SS treatment resulted in marked increases in SOD, MDAHR, DHAR, and GR activities in the chloroplasts of stressed seedlings, and the activity levels were higher than those in S–stressed plants (Figs. 7 and 8). Compared with S treatment, SS treatment also increased the activity of APX in chloroplasts in seedlings of both tomato cultivars. APX activity increased early and declined during the later stages of treatment, with the exception of day 2. This effect was more obvious in cv. JP seedlings (Fig. 8).

**Fig. 9:**
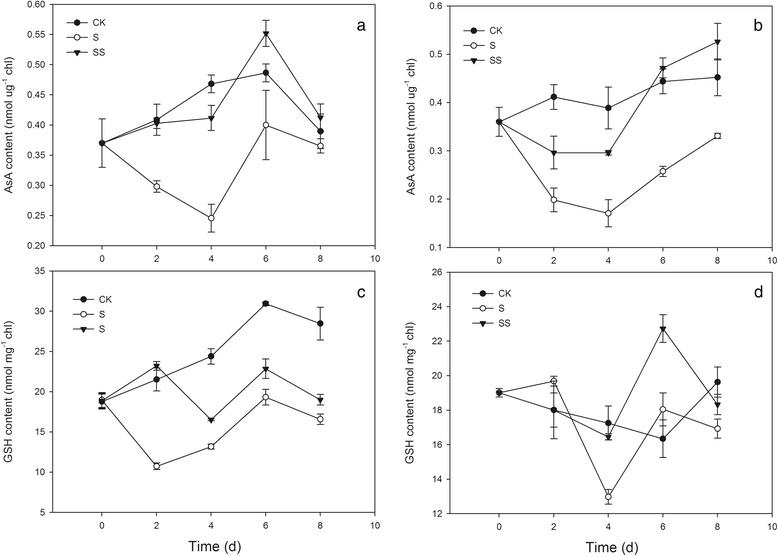
Effect of exogenous Spd on AsA and GSH content in tomato seedlings. CK, 1/2 Hoagland’s solution; S, 75 mM saline–alkaline solution (NaCl: Na_2_SO_4_: NaHCO_3_: Na_2_CO_3_ = 1:9:9:1); SS, sprayed with 0.25 mM Spd and treated with 75 mM saline–alkaline solution. **a** and **c** represent cv. Zhongza No.9; (**b** and **d**) represent cv. Jinpengchaoguan

After salinity–alkalinity stress, the ascorbic acid (AsA) content decreased early and then increased. The AsA concentration in S treatment was lower than that of the control in chloroplasts of both cv. ZZ and cv. JP seedlings (cv. ZZ, 6.21 % versus 47.54 %; cv. JP, 26.86 % versus 56.07 %; Fig. 9). Compared with CK treatment, cv. ZZ seedlings subjected to S treament exhibited significantly lower reduced glutathione (GSH) concent, whereas no obvious change in GSH content was observed in cv. JP seedlings (Fig. 9). SS treatment resulted in a marked increase and similar pattern of change in both the AsA and GSH contents in the chloroplasts of both tomato seedlings. In addition, the extent of the increase in GSH content in cv. ZZ chloroplasts was higher than that in cv. JP chloroplasts, despite on day 0 and day 6 (Fig. 9).

### Effect of Spd on Chloroplast ultrastructure of salinity–alkalinity–stressed tomato seedlings

**Fig. 10:**
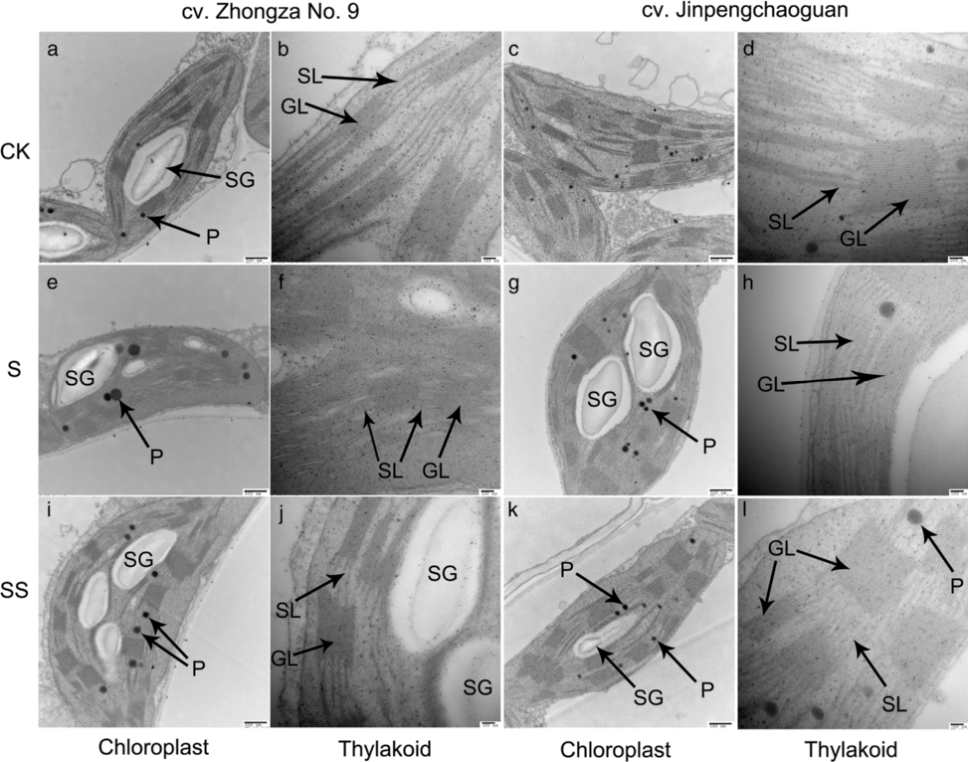
Effect of exogenous Spd on chloroplast ultrastructure in tomato seedlings grown under salinity–alkalinity stress. cv. ZZ, cv. Zhongza No. 9; cv. JP, cv. Jinpengchaoguan; CK, 1/2 Hoagland’s solution; S, 75 mM saline–alkaline solution (NaCl: Na_2_SO_4_: NaHCO_3_: Na_2_CO_3_ = 1:9:9:1); SS, sprayed with 0.25 mM Spd and treated with 75 mM saline–alkaline solution. Data were obtained from the second expanded leaves (numbered basipetally) after salinity–alkalinity treatment for 6 days. SL, stroma lamellae; GL, grana lamellae; SG, starch grains; P, plastoglobuli. Scale bars for chloroplasts and thylakoids are 0.5 and 0.1 μm, respectively. **a** represents chloroplast of CK treated cv. Zhongza No.9; **b** represents thylakoid of CK treated cv. Zhongza No.9; **c** represents chloroplast of CK treated cv. Jinpengchaoguan; **d** represents thylakoid of CK treated cv. Jinpengchaoguan; **e** represents chloroplast of S treated cv. Zhongza No.9; **f** represents thylakoid of S treated cv. Zhongza No.9; **g** represents chloroplast of S treated cv. Jinpengchaoguan; **h** represents thylakoid of S treated cv. Jinpengchaoguan; **i** represents chloroplast of SS treated cv. Zhongza No.9; **j** represents thylakoid of SS treated cv. Zhongza No.9; **k** represents chloroplast of SS treated cv. Jinpengchaoguan; **l** represents thylakoid of SS treated cv. Jinpengchaoguan

Typical spindle chloroplasts were observed in both tomato seedlings under CK treatment, with intact double membranes and a regular arrangement of granal and stromal thylakoids (Fig. 10a–d). Under salinity–alkalinity stress, the chloroplast structures in cv. ZZ seedlings were heavily damaged; the chloroplasts were swollen, the stroma thylakoid stack and grana thylakoid were blurred, and the lamellar structure was destroyed (Fig. 10e and f). The extent of damage to the chloroplast structures of cv. JP seedlings was less than that observed in cv. ZZ seedlings, with some stroma and grana thylakoid structures remaining completely intact (Fig. 10g and h).

The number of plastoglobuli was increased and the plastoglobular volume was abnormally large in S–stressed tomato seedlings of both cultivars, suggesting that the plants were undergoing significant stress. Exogenous Spd alleviated the salinity–alkalinity–induced damage to the chloroplast structure, with a more normal chloroplast ultrastructure observed in SS–treated seedlings. Fewer platoglobuli and lower plastoglobular volume were observed in seedlings subjected to SS treatment versus those subjected to S treatment (Fig. 10i–l).

### Gene expression

**Fig. 11:**
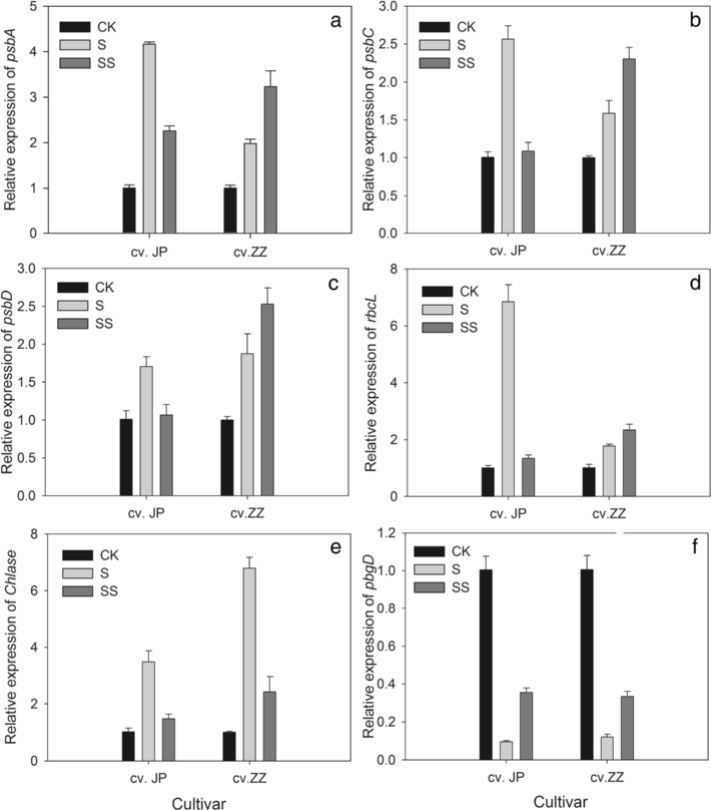
Effect of exogenous Spd on the expression of chlorophyll metabolism enzyme genes. cv. ZZ, cv. Zhongza No. 9; cv. JP, cv. Jinpengchaoguan; CK, 1/2 Hoagland’s solution; S, 75 mM saline–alkaline solution (NaCl:Na_2_SO_4_:NaHCO_3_:Na_2_CO_3_ = 1:9:9:1); SS, sprayed with 0.25 mM Spd and treated with 75 mM saline–alkaline solution. **a**, **c** and **e** represent cv. Zhongza No.9; **b**, **d** and **f** represent cv. Jinpengchaoguan

The relative expression of chloroplast genes (*rbcL*, *psbA*, *psbC*, and *psbD*) and *Chlase* was relatively low in CK-treated  plants (Fig. 11). Salinity–alkalinity stress enhanced the expression of *rbcL*, *psbA*, *psbC*, *psbD*, and *Chlase*, with significantly higher levels of expression of these genes in both tomato cultivars compared with the CK. Under salinity–alkalinity stress, SS treatment resulted in higher levels of *rbcL*, *psbA*, *psbC*, and *psbD* expression in S–stressed cv. ZZ seedlings and lower levels of expression of these genes in S–stressed cv. JP seedlings (Fig. 11). Under salinity–alkalinity stress, SS treatment significantly down–regulated expression of the *Chlase* gene in both cultivars (Fig. 11e), and the extent of this down–regulation was greater in cv. ZZ than in cv. JP seedlings. S treatment also markedly down–regulated expression of the *pbgD* in both cultivars (Fig. 11f), but this change was partly alleviated by exogenous Spd in comparison to S–treatment.

## Discussion

Chl is directly involved in the absorption, transmission, distribution, and transformation of light energy in plants, facilitating the synthesis of organic material from photosynthetic products. In the present study, we found that the Chl a content in stressed cv. JP tomato seedlings was higher than that in control plants from days 2 to 8. The Chl a content in stressed cv. ZZ seedlings and the Chl b and total Chl content in stressed seedlings of both tomato cultivars were lower than in controls after 4 days of stress treatment (Fig. 1). The Chl content increased during the early stress period (days 0–4) and declined during the later stress period (days 4–8), consistent with the report of Romero et al. [20]. These results suggest that transient salinity–alkalinity stress stimulates the accumulation of Chl, but as the duration of stress increases, the Chl content declines.

Chl content is affected by the rates of Chl synthesis and degradation [5]. The Chl biosynthesis pathway in higher plants is complex, mediated by more than 17 enzymes [21]. The conversion of glutamic acid into Mg–proto IX occurs in the chloroplast, and the conversion of Mg–proto IX into Chl b occurs in the thylakoid membrane [22]. Disruption of any of these reaction steps may result in significant accumulation of intermediates produced in steps prior to the point of disruption and a significant decrease in the amount of products produced in subsequent steps. Chen et al. found that seawater stress hinders the transformation of PBG to URO III in spinach [23]. Wang et al. suggested that UV–B disrupts Chl synthesis at the point of ALA conversion to PBG [24]. This difference may be crop– or cultivar–specific [25]. In the present study, salinity–alkalinity stress induced the over–accumulation of ALA, PBG, and URO III in seedlings of both tomato cultivars throughout the experimental period (Figs. 2 and 3). Salinity–alkalinity stress also caused an increase in the Proto IX content from days 0–2 in cv. ZZ seedlings and days 0–4 in cv. JP seedlings and an increase in the contents of Mg–proto IX and Pchl in both tomato cultivars from days 0–4, relative to the controls. However, between days 6 and 8, levels of Proto IX, Mg–proto IX and Pchl declined and were significantly lower than in controls (Fig. 4). These results indicated that salinity–alkalinity stress disrupted Chl synthesis at the step of URO III conversion into Proto IX, which can be attributed to damage to the thylakoid membrane [26]. These results also indicated that salinity–alkalinity stress upset the Chl biosynthesis balance differently in cv. ZZ and cv. JP seedlings.

An increase in Chl content could also be due to a decrease in Chl degradation or to an increase in Chl synthesis. In the present study, stress led to an increase in Chl content between days 0 and 4 and a decrease in Chl content thereafter, whereas more severe salinity–alkalinity stress stimulated the activity of Chlase over time (Fig. 5). These results indicate that Chlase accelerates the degradation of Chl in tomato during long–term salinity–alkalinity stress, which could explain in part why long–term stress leads to disorganization of chloroplasts followed by increased contact of Chl with Chlase, in turn leading to an increase in Chlase activity. Maintenance of the structural integrity of chloroplasts is necessary for the conversion of light energy during photosynthesis. Fang et al. hypothesized that chloroplast degradation is responsible for the decrease in Chlase activity [27]. Further analysis of the ultrastructure of chloroplasts in the present study indicated that salinity–alkalinity stress induced destruction of the chloroplast envelope and increased the number of plastoglobuli and aberrations in the thylakoid membrane (Fig. 10). These results demonstrate that although Chl degradation is undoubtedly responsible at least in part for the decline in Chl content, during severe stress this process is not dependent on the activity of Chlase, suggesting that an alternative pathway must be involved. The decrease in Chl content may be attributed to molecular–level Chl damage, resulting in decrease in the efficiency of light energy absorption and transmission in the chloroplast.

Polyamines exert positive effects on photosynthetic efficiency under stress conditions due to their acid–neutralizing and antioxidant properties, as well as their membrane– and cell wall–stabilizing activity [28]. PAs with a high net positive charge can stabilize photosystem II (PSII) proteins such as D1 and D2 under photo–inhibition conditions [29]. PAs binding to membrane proteins may stabilize the protein structure during stress and consequently preserve photosynthetic activity. Exogenous Spd alleviated the negative effects of salinity–alkalinity stress on Chl content (Fig. 1) and the damage to the chloroplast photosynthetic apparatus, resulting in a more normal chloroplast ultrastructure in Spd–treated plants (Fig. 10). These results indicate that exogenous Spd may play a protective role in chloroplasts, ensuring that a sufficient supply of enzymes are available for conversion of URO III to Proto IX, thus promoting Chl synthesis and enhancing Chl a and Chl b levels in tomato seedlings grown under salinity–alkalinity stress. Under salinity–alkalinity stress, exogenous Spd reduced the stress–induced increase in Chlase activity and the ALA, PBG, and URO III levels in both tomato cultivars; the URO III content in SS–treated cv. ZZ and cv. JP seedlings declined on days 4 and 2, respectively (Figs. 2 and 3), suggesting that the effect of Spd on stress–induced changes in Chl synthesis differs between cultivars, with the effect of Spd apparent earlier in the more tolerant cultivar (cv. JP) than in the more sensitive cultivar (cv. ZZ). Exogenous Spd also attenuated the increase in Chlase activity after day 4 of the stress period, maintaining the Chl a, Chl b, and total Chl levels, contrary to the trend observed in stressed plants not treated with Spd. These results indicate that exogenous Spd decreases the accumulation of URO III by promoting the conversion of Proto IX to Chl, thus overcoming the stress–associated blockade of URO III conversion to Proto IX. These effects may be attributed to the stabilization of chloroplast structure by Spd, which ensures that sufficient enzymes are available for conversion of URO III to Proto IX, thereby promoting Chl synthesis.

The *psbA* gene plays a critical role in the *de novo* synthesis of D1 protein and the repair of photo–damaged PSII components [30]. A previous study reported that salinity stress reduces the transcript levels of several chloroplastic genes (*rbcL*, *psbA*, *psbB*, and *psbE*) [31]. In the present study, salinity–alkalinity stress led to increases in the levels of transcripts of the *rbcL*, *psbA*, *psbC*, and *psbD* genes. This increase was more pronounced in cv. JP than cv. ZZ (Fig. 11). Our results show that exogenous Spd leads to down–regulation of the expression of *rbcL*, *psbA*, *psbC*, *psbD* and *Chlase* and the maintenance of near–normal transcript levels in cv. JP, in agreement with the results of Chattopadhayay et al. [31]. However, exogenous Spd up–regulation of the expression of *rbcL*, *psbA*, *psbC*, *psbD* and *pbgD* in cv. ZZ, which may be one of the reasons for cv. JP was more tolerant to salinity–alkalinity stress than cv. ZZ. The expression of *pbgD* was down–regulated and that of *Chlase* was up–regulated under salinity–alkalinity stress. However, the down–regulation of *pbgD* and up–regulation of *Chlase* was alleviated by exogenous Spd (Fig. 11). These results provide conclusive evidence that exogenous Spd has a positive effect in preventing the loss of Chl in stressed plants by promoting Chl synthesis and alleviating Chl degradation.

Of all plant organelles, chloroplasts seem to be the most sensitive to salt stress and are the major source of ROS. ROS such as O_2_^•−^, hydroxyl ions (OH^−^), and H_2_O_2_, may oxidize proteins, lipids, and nucleic acids. This may result in abnormalities at the cellular level when plants are exposed to environmental stresses [32]. This may particularly affect photosystem I through the oxidation of iron oxide reducing protein [32]. ROS can be generated by the direct transfer of the excitation energy from Chl to produce singlet oxygen or by oxygen reduction through the Mehler reaction in the chloroplasts, which leads to membrane lipid peroxidation [33]. Environmental stresses such as high salinity aggravate photo–inhibition and over a long period may induce photo–oxidization, resulting in accumulation of ROS in chloroplasts. Over–accumulation of ROS leads to enzyme inactivation, pigment decolorization, protein degradation, and lipid peroxidation, ultimately inhibiting plant growth. In the present study, seedlings of two tomato cultivars exhibited increased chloroplast ROS accumulation under salinity–alkalinity stress (Fig. 6). In plants, antioxidant systems readily scavenge ROS to protect cells from oxidative damage. However, under stressful conditions, the production of ROS may overwhelm the capacity of the antioxidant system, thereby resulting in oxidative stress symptoms [34]. In an efficiently functioning antioxidant system, a high level of antioxidant enzyme activity and high levels of non–enzymatic components are maintained. In the present study, salinity–alkalinity stress led to enhanced chloroplast SOD, GR, APX, DHAR, and MDHAR activities in seedlings of both tomato cultivars (Figs. 7 and 8). An increase in MDHAR activity can provide reducing equivalents for APX, which can maintain the AsA–GSH cycle. MDHAR activity was higher than DHAR activity in our study (Fig. 8), indicating that AsA regeneration may act through MDHAR reduction to monodehydroascorbate. However, the ability of DHAR and MDHAR to catalyze AsA regeneration is limited, resulting in reduced AsA content. In the present study, AsA regeneration under salinity–alkalinity stress was primarily driven by APX in cv. ZZ and by MDHAR in cv. JP. Glutathione acts as a substrate for glutathione peroxidase and is considered the critical component of the AsA–GSH cycle for maintaining intracellular defenses against ROS–induced oxidative damage [35, 36]. The increase in GR activity directly promotes conversion of oxidized glutathione to GSH, which eliminates H_2_O_2_ and reduces the accumulation of ROS in chloroplasts [37].

PAs are also well known for their positive effects on photosynthetic efficiency under stress conditions due to their acid–neutralizing and antioxidant properties. Spd contains highly protonated amino and imino groups and may conjugate with other negatively charged organic molecules such as nucleic acids, proteins, and phospholipids. Such binding is important for the stabilization of the thylakoid membranes and prevention of the hydrolysis of photosynthetic proteins [38]. In the present study, application of exogenous Spd also resulted in suppression of physiological damage associated with salinity–alkalinity stress, as shown by the lower MDA content and O_2_^•−^ generation rate (Fig. 6), thus confirming previous observations that exogenous PAs significantly improve the physiological status of stressed plants [17, 39]. Moreover, the positive influence of Spd on MDA content and O_2_^•−^ generation rate differed between cultivars, possibly indicating that Spd has a more beneficial effect on sensitive cultivars grown under stress conditions. In the present study, application of exogenous Spd significantly increased the activities of the ROS–scavenging enzymes SOD, APX, and GR in the chloroplasts of salinity–alkalinity–stressed tomato seedlings. Moreover, exogenous Spd induced the synthesis of antioxidant metabolites that provide additional capability to neutralize the toxic effects of ROS generated during salt stress [40]. We observed that Spd increased the contents of AsA and GSH in chloroplasts, which enhanced the salinity tolerance of the photosynthetic apparatus. The contents of antioxidant metabolites and activities of enzymes in salinity–alkalinity–stressed chloroplasts were enhanced by Spd application, consistent with the observed effects of Spd in reducing the O_2_^•−^ generation rate and MDA content in tomato seedling chloroplasts. These results showed that Spd alleviates chloroplast membrane injury resulting from salinity–alkalinity stress through an increase in ROS scavenging, indicating that Spd may protect PS II from oxidative stress.

## Conclusions

In conclusion, the two tomato cultivars examined in the present study exhibited different response capacities to salinity–alkalinity stress. Exogenous Spd is effective in triggering protection against cellular and macromolecular damage in tomato seedlings during salinity–alkalinity stress, probably by maintaining the structural integrity of chloroplasts and alleviating salinity–alkalinity–induced oxidative damage, most likely through regulation of Chl metabolism and enzymatic and non–enzymatic antioxidant systems in the chloroplasts. Exogenous Spd alleviates the down–regulation of *pbgD* and up–regulation of *Chlase* expression under stress conditions, which may promote an increase in Chl content. Exogenous Spd also exhibits positive effects in maintaining the expression of the *rbcL* and *psbA* genes. Exogenous Spd decreases the accumulation of URO III and promotes the conversion of Proto IX to Chl, thus alleviating the stress–associated blockade of URO III conversion to Proto IX. This effect was more pronounced in the sensitive cultivar than the tolerant cultivar and earlier in the more tolerant cultivar than in the more sensitive cultivar.

## Methods

### Plant culture, salinity–alkalinity stress, and sample collection

Six true–leaves–old tomato (*Solanum lycopersicum* L.) seedlings of cv. JP (tolerant to salinity–alkalinity stress) and cv. ZZ (sensitive to salinity–alkalinity stress) were initially grown in one–half–strength Hoagland’s solution in an environmentally controlled greenhouse, as described by Zhang et al. [2]. After 7 days of pre–culture under controlled conditions, the seedlings were treated with 75 mM salinity–alkalinity solution (molar ratio of NaCl:Na_2_SO_4_:NaHCO_3_:Na_2_CO_3_ = 1:9:9:1) and the foliage was sprayed with 0.25 mM Spd. The experimental plots included three treatments: (a) CK, half–strength Hoagland’s nutrient solution + 0 mM Spd; (b) S, 75 mM salinity–alkalinity + 0 mM Spd; and (c) SS, 75 mM salinity–alkalinity + 0.25 mM Spd.

The containers were arranged in completely randomized blocks, with four replicates per treatment. The nutrient solutions were renewed every 2 days. After 0, 2, 4, 6, and 8 days of stress treatment at the final concentration, the second fully expanded leaf from the top of each plant was used to analyze chlorophyll content, chlorophyll metabolism, and antioxidant enzymes in the chloroplasts. The relative expression of genes in the tomato seedlings was analyzed after 4 days of treatment. Changes in chloroplast ultrastructure were evaluated after 6 days of treatment.

### Determination of Chl precursors

Chl a, Chl b, and Chl (a + b) levels were estimated following the method of Holden [41]. Proto IX, Mg–proto IX, and Pchl were extracted using a mixture of acetone:ammonia (1 %) (4:1) and the contents were determined based on the absorbance of the extracts at 575, 590, and 628 nm, respectively [42]. Fresh leaves were homogenized on ice in Tris–HCl (pH 7.2), the homogenate was centrifuged at 5000 × *g* for 15 min at 4 °C, and the URO III content in the supernatant was determined by the method of Bogorad [43]. For PBG determination, leaf samples were homogenized with Tris–HCl buffer (pH 8.0) containing 100 mM Tris and 50 mM mercaptoethanol, and the homogenate was centrifuged at 8000 × *g* for 15 min at 4 °C. Next, 2 mL of the supernatant or standard solution was mixed with 2 mL of freshly prepared Ehrlich’s reagent, and after 30 min, the mixture was used to determine the PBG content at 555 nm according to the method of Bogorad [43]. For the determination of ALA, fresh leaves were homogenized in acetic sodium buffer (pH 4.6), the homogenate was extracted in a boiling water bath for 15 min and then centrifuged at l0,000 × *g* for 20 min at 4 °C, and the ALA content in the resulting supernatant was determined according to the method of Richard [44]. The ALA content was based on reference to an ALA–HCl standard (Sigma–Aldrich, St. Louis, MO, USA).

### Assay of Chlase activity

Samples of frozen tomato leaves were ground on ice with pre–chilled acetone (−20 °C). The homogenate was centrifuged at 3000 × *g* for 5 min at 4 °C, and the pellet was collected. The cold acetone extraction procedures were repeated three times in the same manner to remove all traces of Chls and carotenoids. The resulting acetone powder was dried under nitrogen gas and stored at −20 °C until use [45]. The acetone powder was homogenized in 5 mL of extraction buffer (50 mM potassium phosphate [pH 7.0], 50 mM KCl, and 0.24 % Triton X–100) for 1 h at 30 °C in a water bath. After centrifugation at 12,000 × *g* for 10 min at 4 °C, the supernatant was used for the enzyme assay. A total of 4 g of spinach fresh mass (FM) were homogenized in 40 mL of acetone:water (80:20, vol/vol) at 4 °C using an omnimixer. The suspension was centrifuged at 9000 × *g*, and 40 mL of petroleum ether was added to the supernatant to extract the Chls. The ether was then evaporated under N_2_, and the extracted Chls were dissolved in 4–5 mL of acetone. To assay Chlase activity, 1 mL of supernatant, 0.5 mL of reaction buffer (50 mM sodium phosphate [pH 7.0] and 0.24 % Triton X–100), and 2 mL of Chl substrate were mixed, incubated for 30 min at 40 °C, and then poured into 5 mL of hexane:acetone (7:3) pre–cooled in ice water. The resulting mixture was stirred vigorously until an emulsion formed, and then centrifuged at 6000 × *g* for 5 min at 4 °C. The upper phase of the resulting supernatant contained the remaining Chl, whereas the lower phase contained the chlorophyllide. Chlase activity was monitored by measuring the absorbance of the lower phase at 663 nm. Enzyme activity was expressed as the increment of optical density at 663 nm per minute under the test conditions employed [46].

### Transmission electron microscopy of chloroplasts

The second fully expanded leaves from the top of the plants were randomly selected for electron microscopic examination. The leaf samples were sectioned and then examined using a HITACHI HT7700 transmission electron microscope according to the method described by Hu et al. [2].

### Isolation of intact chloroplasts, MDA and O_2_^•−^ generation rate measurements

Intact chloroplasts were isolated using the method described by Shu et al. [19]. MDA was measured according to the method of Xu et al. [47]. The O_2_^•−^ generation rate was determined according to the method of Elstner and Heupel [48] with a slight modification. The 100 μL chloroplast supernatants were added into 200 μL of ice–cold PBS buffer (65 mM, pH 7.8) and 300 μL hydroxylamine chlorhydrate, placed at 30 °C for 20 min, extracted by diethyl ether, and then centrifuged at 3000 × *g* for 5 min at room temperature. Three hundred microliters of the extract was added to a tube, and 500 μL 17 mM sulfanilamide and 500 μL 7 mM α–naphthylamine were added. The mixture was then placed at 30 °C for another 20 min before mixing with 2.25 mL pure ether. The absorbance was measured at 530 nm and the O_2_^•−^ generation rate was calculated from a NaNO_2_ standard curve.

### Extraction of chloroplast antioxidant enzymes and antioxidants

A 3–mL aliquot of Chl–containing supernatant was mixed with 3 mL of ice–cold HEPES buffer (25 mM, pH 7.8) containing 0.2 mM ethylene diamine tetraacetic acid and 2 % (w/v) poly vinyl pyrrolidone. The mixture was then centrifuged at 4 °C at 12,000 × *g* for 20 min. The resulting supernatant was used to assay the antioxidant enzyme activity and determine the content of antioxidants (AsA and GSH).

### Measurement of SOD, APX, GR, MDHAR, and DHAR activities

SOD activity was assayed by monitoring SOD–mediated inhibition of the photochemical reduction of nitro blue tetrazolium (NBT) [49]. One unit of SOD activity was defined as the amount of enzyme required for 50 % inhibition of the reduction of NBT, as monitored at 560 nm.

APX activity was assayed using the method of Nakano and Asada by monitoring the ascorbate oxidation rate at 290 nm [50].

GR activity was measured by tracking NADPH oxidation by monitoring the decrease in absorbance at 340 nm over 3 min [51].

The activities of MDHAR and DHAR were assayed according to the method described by Zhang et al., with a slight modification [52]. MDHAR activity was assayed at 340 nm in a 1–mL sample containing 50 mM HEPES–KOH (pH 7.6), 25 mM AsA, 1 mM NADH, 0.5 units of ascorbate oxidase, and 50 μL of enzyme extract. DHAR activity was assayed at 265 nm in a 2.9–mL sample containing 100 mM HEPES–KOH (pH 7.6), 25 mM reduced GSH, 2 mM dehydroascorbate, and 50 μL of enzyme extract.

Protein was determined according to the method of Bradford, using bovine serum albumin as a standard [53].

### Determination of AsA and GSH content

Ascorbate was determined according to the method of Shu et al. [19], with a minor modification. The reaction mixture contained 200 μL of 5 % trichloroacetic acid, 100 μL of 0.4 % H_3_PO_4_–ethanol, 100 μL of 0.03 % FeCl_3_–ethanol, 200 μL of 0.5 % BP–ethanol, and 300 μL of extract. The sample was incubated at 40 °C for 1 h, after which the absorbance was measured at 534 nm. AsA content was calculated based on an ascorbic acid standard curve.

GSH content was assayed as described by Li and Cheng [54]. GSH was determined by subtraction of oxidized glutathione from total glutathione.

### Expression of chlorophyll metabolism enzyme genes

Total RNA was extracted from tomato leaves using an E.Z.N.A.® Plant RNA Kit (Omega Bio–Tek, Doraville, GA, USA) according to the manufacturer’s instructions. The total RNA was then reverse–transcribed using a PrimeScript^TM^ RT reagent kit with gDNA Eraser (Takara, Shiga, Japan) in a 20–μL reaction mixture containing 1 μL of total RNA from each individual sample. Real–time PCR was performed on a CFX96™ real–time PCR cycler (Bio–Rad, Hercules, CA, USA) and a SYBR Premix Ex Taq (TliRNaseH Plus) Kit (Takara). Initial denaturation at 95 °C for 30 s was followed by 40 cycles of 95 °C for 5 s, 58 °C for 30 s, and a melting curve of 65–95 °C. Primers for the actin gene were used as an internal control. Primers for *psbA* and actin were designed as described by Wu et al. [55]. Primers for the *pbgD* and *Chlase* genes were designed using Primer3, version 4.0.0 (website software), with the primer length set at 20 − 24 bp; melting temperature of 58 − 62 °C; CG content, 30 − 70 %; and product size, 150–250 bp. All samples were analyzed three times.

### Statistical analysis

All experiments were performed with at least three replicates. Data represent the mean ± SE. Data were analyzed with SAS 9.0 software (SAS Institute, Cary, NC, USA) using Duncan’s multiple range tests, with *P* < 0.05 defining significance. Different letters in table indicate significant differences between means.

## Abbreviations

ALA: δ–aminolevulinic acid
APX: ascorbateperoxidase
AsA: ascorbic acid
Chl: chlorophyll
Chlase: chlorophyllase
CK: control
cv. JP: cultivar Jinpengchaoguan
cv. ZZ: cultivar Zhongza No. 9
DHAR: dehydroascorbate reductase
GL: grana lamellae
GR: glutathione reductase
GSH: glutathione
H_2_O_2_: hydrogen peroxide
MDA: malondialdehyde
MDHAR: monodehydroascorbate reductase
Mg–proto IX: Mg–protoporphyrin IX
O_2_^–⋅^: superoxide radical
OH^−^: hydroxyl ion
P: plastoglobuli
PAs: polyamines
PBG: porphobilinogen
*pbgD*: porphobilinogen deaminase
Pchl: protochlorophyll
Proto IX: protoporphyrin IX
PSII: photosystem II
ROS: reactive oxygen species
S: salinity–alkalinity–stress treatment
SG: starch grains
SL: stroma lamellae
SOD: superoxide dismutase
SS: salinity–alkalinity plus Spd treatment
URO III: uroorphyrinogen III
